# Prevalence of Osteoporosis Among Patients Visiting Primary Health Clinics in Al-Ahsa, Saudi Arabia, From 2021 to 2024

**DOI:** 10.7759/cureus.86900

**Published:** 2025-06-28

**Authors:** Wafaa M Alshaikh, Zainab Almurayhil, Qassem Al-jaber

**Affiliations:** 1 Family Medicine, Al-Ahsa Health Cluster, Al-Ahsa, SAU

**Keywords:** al-ahsa, dexa, osteoporosis, saudi arabia, screening

## Abstract

Background

Osteoporosis, recognized as a debilitating condition, significantly affects skeletal health and overall quality of life by increasing the risk of fractures and functional impairments. This retrospective cross-sectional study aims to evaluate the prevalence of osteoporosis and explore associated demographic factors among individuals who underwent dual-energy X-ray absorptiometry (DEXA) screening between 2021 and 2024 in primary healthcare (PHC) centers in Al-Ahsa, Saudi Arabia.

Materials and methods

This retrospective cross-sectional study was conducted at primary healthcare centers in Al-Ahsa, Saudi Arabia. The study used data from patients' medical records collected from 2021 to 2024. After obtaining approval from the Research Ethics Committee of Prince Saud Bin Jalawi Hospital, we collected data from the medical records, including age, sex, body mass index (BMI), and DEXA scan results. The inclusion criteria included all patients who underwent DEXA scans during the specified period, excluding those with incomplete data. Statistical analysis included descriptive statistics to assess the osteoporosis prevalence and treatment rates. Independent sample t-tests were used to compare mean age and BMI between groups, while chi-square tests were used to evaluate associations between categorical variables, such as sex and osteoporosis diagnosis.

Results

A total of 5,768 valid and clean records were included in the final analysis. The cohort comprised 66.8% women (n=4,357) and 33.2% men (n=2,169). The mean age was 64.7±8.2 years (range: 40-104), and the mean BMI was 30.5±6.0 kg/m². Most participants were from the eastern sector (48.7%), followed by the middle (25.3%), southern (16.5%), and northern (9.6%) sectors. The highest sector with a T-score equal to -2.5 was the eastern sector, around 32.8%, followed by the northern, middle, and southern sectors, around 32.2%, 30.4%, and 28.7%, respectively. Among the study population, 31.54% had a T-score of less than or equal to -2.5, indicating osteoporosis. When analyzed by sex, the condition was present in 36.7% of women and 21.16% of men, reflecting the prevalence within each sex group. Compared with international benchmarks, the total treatment rate observed in our study (11.49%) was notably low. Patients with the most severe T-scores (equal to -2.5 or less) exhibited the lowest mean BMI, while those with higher T-scores (between -1.0 and -2.5 and equal to -1.0 or higher) had progressively higher mean BMI values.

Conclusion

The findings highlight significant disparities in osteoporosis prevalence and treatment uptake between sexes, with women showing a higher prevalence (36.7%) but equally low treatment rates compared to men (11.48% versus 11.53%). Regional variations were also observed, with the eastern sector having the highest proportion of individuals with osteoporosis. Emphasizing the need for targeted interventions may include strengthening community-based screening programs, clinician education on guideline-based treatment, and incorporating osteoporosis risk assessment into routine check-ups. Policy changes should prioritize expanding insurance coverage for DEXA scans and ensuring equitable access to pharmacologic therapy for at-risk populations.

## Introduction

Osteoporosis is a significant health concern that affects millions of people globally, as recognized by the World Health Organization (WHO) in 1994 [1]. Characterized by diminished bone mineral density (BMD), osteoporosis increases the risk of fractures and morbidity and mortality rates [1]. According to the World Health Organization (WHO) criteria, this condition is diagnosed when the BMD T-score falls below -2.5 [2]. Osteoporosis is classified into two main types: primary and secondary. Primary osteoporosis often affects postmenopausal women and those over 70 years of age, owing to natural aging processes and hormonal changes [3]. In contrast, secondary osteoporosis results from other systemic conditions such as the chronic use of glucocorticoids, endocrine disorders, malignant neoplasms, and additional factors [3]. During the menopausal transition, women experience a marked decrease in bone mineral density, with an average reduction of approximately 10%. Nearly half of these women may experience accelerated BMD loss of up to 20% over 5-7 years of menopause [4]. Osteoporosis is associated with both non-modifiable and modifiable risk factors. Non-modifiable factors include age, sex, and genetic predisposition, whereas modifiable factors include dietary insufficiencies, the lack of physical activity, smoking, excessive alcohol consumption, and the use of certain medications [5]. Women are often at a higher risk of premature menopause and other related factors [6]. A recent study in Turkey illustrated that women aged 18-49 years who smoke, have lighter skin tones, or have a family history of osteoporosis face an elevated risk of developing the disease [7]. Engaging in regular physical activity can positively influence BMD, while obesity seems to offer some protective benefits, given the inverse relationship between body mass index (BMI) and BMD [8]. Dual-energy X-ray absorptiometry (DEXA) remains the gold standard for measuring the BMD at various skeletal sites. A BMD T-score of -2.5 at the femoral neck, lumbar spine, or distal radius indicates osteoporosis [5]. Moreover, individuals who suffer low-trauma spine or hip fractures, irrespective of BMD, or those with a T-score of -1.0 to -2.5 accompanied by a fragility fracture are diagnosed with osteoporosis [5].

Screening recommendations vary by region and risk profile. In the United States, the US Preventive Services Task Force (USPSTF) advises DEXA screening for all women aged ≥65 years and younger with a history of fractures and additional risk factors [9]. Conversely, there are no routine screening recommendations for men [10]. In Saudi Arabia, it is recommended that comprehensive osteoporosis screening be conducted for both men and women aged 60 years and above. This recommendation is informed by local data indicating a high and variable prevalence of osteoporosis across different regions within the country. Additionally, it is particularly advised for women aged 40 years and older who have experienced low-trauma fractures or exhibit specific risk factors [10]. Osteoporosis remains a critical health concern in Saudi Arabia, with prevalence rates varying from 23.4% to 39.5% among adults, significantly affecting both healthcare and economic resources [11-13]. In Riyadh, a retrospective study involving 1,302 patients, predominantly women with an average age of 68.26 years, indicated a femoral osteoporosis prevalence of 8.2% and a lumbar spine prevalence of 11.8% [14]. These regional differences in prevalence may be influenced by variations in population age structure, access to DEXA screening services, diagnostic practices, and public awareness of osteoporosis. Additionally, differences in referral patterns and healthcare utilization between urban and semi-urban settings may contribute to the observed disparities. Conversely, research in Alkhobar at King Fahd Hospital analyzed data from 301 patients from 2018, revealing a high osteoporosis prevalence of 63.6% in men and 52.8% in women, with an average age of 65.2 years for men and 62.9 years for women [12].

These studies underscore the critical gender- and age-related differences in osteoporosis prevalence, necessitating targeted preventive and screening measures across the nation, especially in regions such as Al-Ahsa. Therefore, measuring the prevalence of osteoporosis in Al-Ahsa, the eastern province of Saudi Arabia, is crucial for future preventive measures.

## Materials and methods

This study aimed to evaluate the prevalence of osteoporosis among individuals who underwent dual-energy X-ray absorptiometry (DEXA) screening between 2021 and 2024 at primary healthcare (PHC) centers in Al-Ahsa, Saudi Arabia. This retrospective cross-sectional study analyzed 10,695 individuals who underwent DEXA scans during this period, using the entire dataset for a comprehensive analysis to enhance the accuracy of the results and better represent the population. A stratified random sampling technique was employed. The stratification variables encompassed geographic sectors, determined by the locations of primary healthcare centers in the eastern, northern, central, and southern regions. These variables are crucial for ensuring demographic and regional representation within the Al-Ahsa population. Inclusion criteria were as follows: all patients had undergone a DEXA scan within this timeframe. Patients with incomplete data, missing one or more of the variables age, sex, BMI, or DEXA scan results, were excluded from the study. Data were collected by leveraging medical records to gather data on age, sex, body mass index (BMI), and DEXA scan results. We aimed to uncover how various factors influence osteoporosis prevalence and treatment effectiveness. This methodological approach ensured that our analysis accurately reflected the osteoporosis landscape within the PHC population.

In our research, we applied a variety of statistical methods to scrutinize the data obtained from primary healthcare centers in Al-Ahsa from 2021 to 2024. To assess the prevalence of osteoporosis and the percentage of patients receiving treatment for osteoporosis, we used descriptive statistics, calculating percentages and proportions to gain insights into these factors. To compare the age at which osteoporosis was diagnosed between sexes, we used an independent sample t-test to verify whether the data met the criteria for normal distribution and variance homogeneity. Osteoporosis was diagnosed based on DEXA-derived T-scores, in accordance with WHO criteria (T-score of less than or equal to -2.5). The chi-square test was used to assess significant associations between osteoporosis status and categorical variables such as sex and treatment uptake. Additionally, to evaluate the effect of BMI on the probability of osteoporosis diagnosis, we performed an independent sample t-test to compare the average BMI values of individuals with and without osteoporosis, ensuring that the data conformed to the assumptions of the test. We ensured that the data on the master database with participant identifiers were established and securely stored in password-protected applications, such as Dropbox (Dropbox, Inc., San Francisco, CA) and Google Drive (Google, Inc., Mountain View, CA), with access restricted to the investigators. Ethical approval for this study was obtained from the Research Ethics Committee of Prince Saud Bin Jalawi Hospital, Ministry of Health, Al-Ahsa, Saudi Arabia (approval number: H-05-HS-135).

## Results

**Figure 1:**
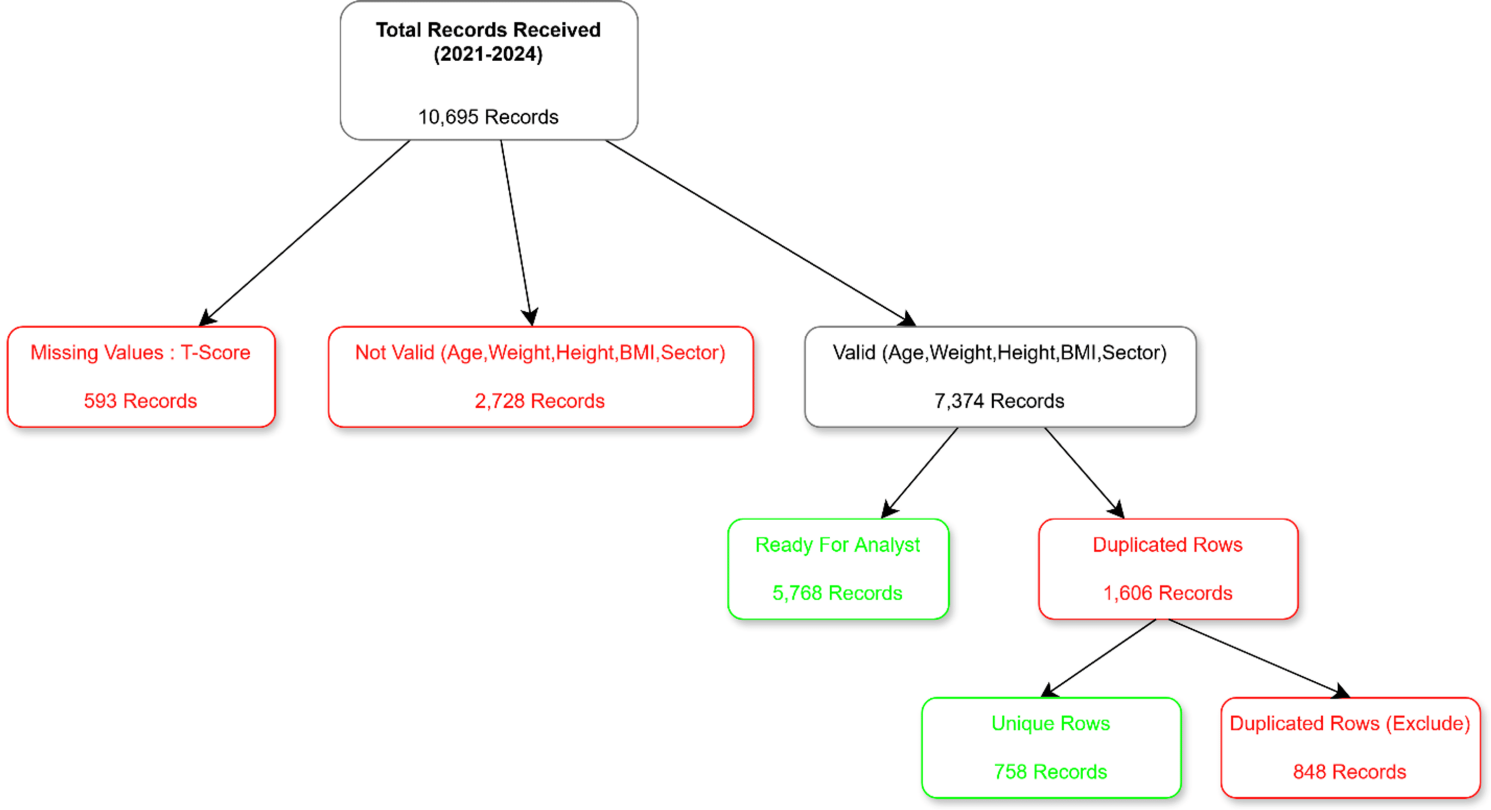
A total of 10,695 records received Only 5,768 valid and clean records were included in the final analysis BMI: body mass index

A total of 10,695 DEXA records collected between 2021 and 2024 were reviewed. After excluding records with missing T-scores (n=593), invalid demographic data (n=2,728), and duplicate entries (n=1,606), 5,768 valid and cleaned records were included in the final analysis (Figure 1). The cohort comprised 66.8% women (n=4,357) and 33.2% men (n=2,169). The mean age was 64.7±8.2 years (range: 40-104), and the mean BMI was 30.5±6.0 kg/m². Most participants were from the eastern sector (48.7%), followed by the middle (25.3%), southern (16.5%), and northern (9.6%) sectors (Table 1).

**Table 1:** The values (count and percent) for each group

Variable	Group	Count (N)	Percent (%)
Gender	Female	4,357	66.76
	Male	2,169	33.24
Sector	Middle	1,653	25.33
	South	1,074	16.46
	East	3,175	48.65
	North	624	9.56
T-score	Between -1.0 and -2.5	2,760	42.29
	Equal to -1.0 or higher	1,708	26.17
	Equal to -2.5 or lower with fracture	2,058	31.54
Year	2021	1,536	23.54
	2022	1,609	24.66
	2023	2,048	31.38
	2024	1,333	20.43
Treatment	No	5,776	88.51
	Yes	750	11.49

**Figure 2:**
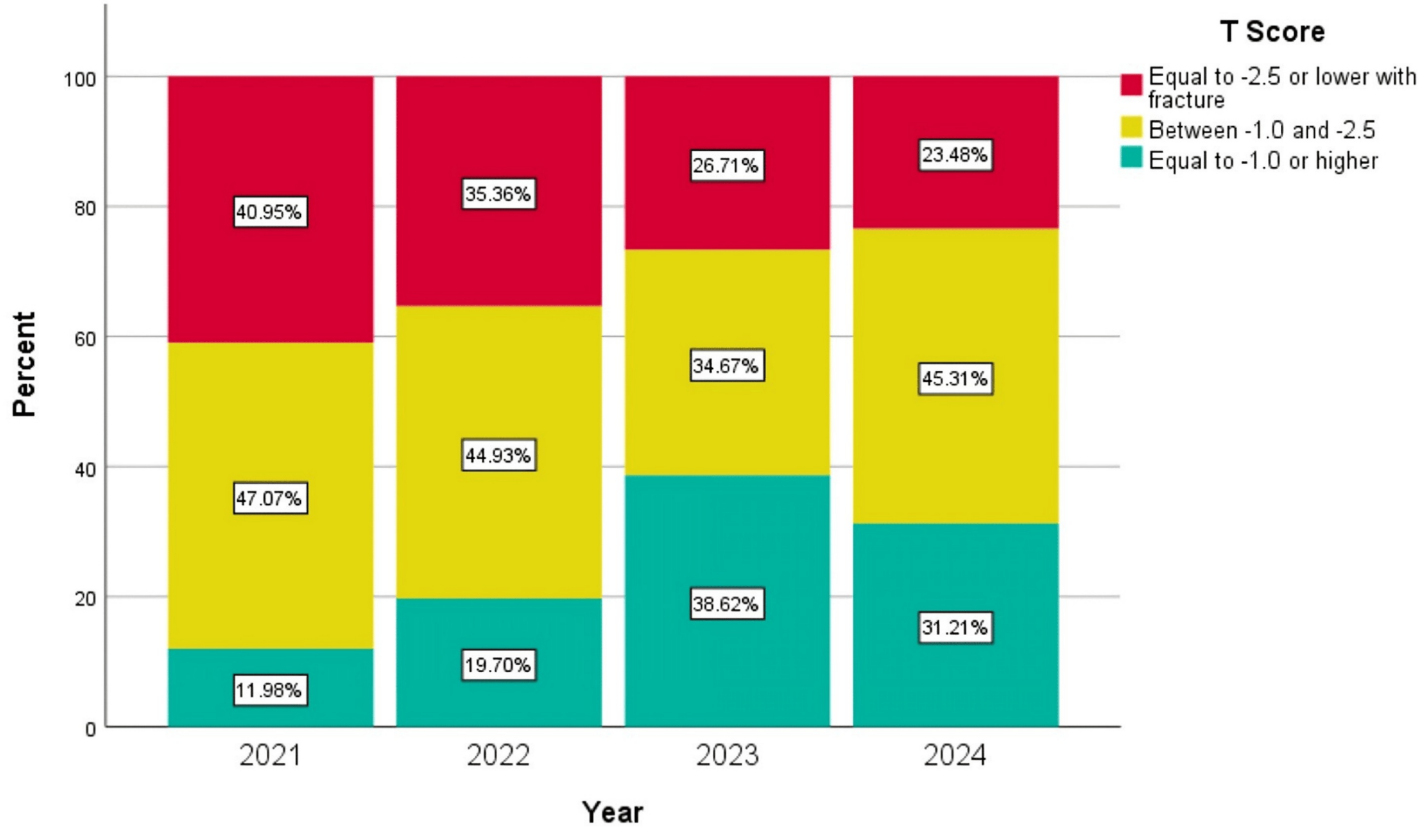
Yearly compression of osteoporosis prevalence The analysis yielded a significant positive correlation between year and osteoporosis classification, with Spearman's correlation coefficient of rs=0.202 and a significance level of p<0.01. This indicates a weak-to-moderate positive association, suggesting that as the years progressed, osteoporosis severity tended to decrease, with more patients in the higher T-score categories over time

**Figure 3:**
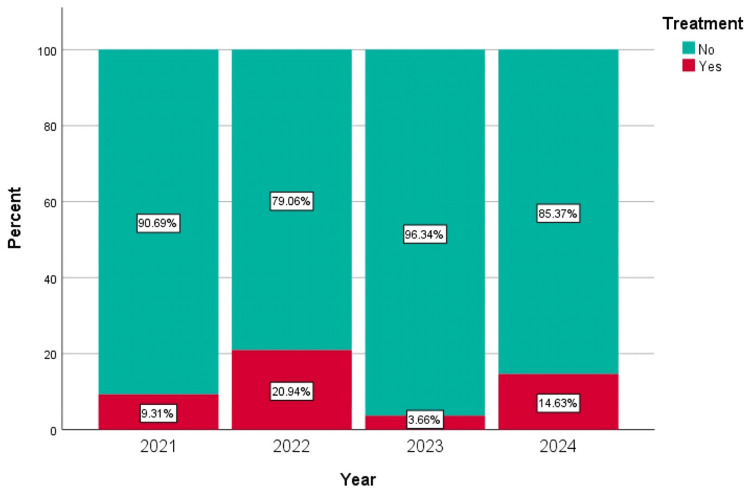
Treatment analysis across years The on-treatment variable was defined as follows: "Yes" for patients with a T-score of -2.5 or lower accompanied by a fracture, who are listed on the treatment list, and "No" for all other patients. The analysis produced Spearman's correlation coefficient of rs=-0.025 with a significance level of p=0.046. This result indicated a very weak negative correlation between the year of screening and treatment status. However, the relationship was statistically significant (p<0.05), suggesting that treatment status varied significantly with the year of screening

A significant positive correlation was observed between years of screening and osteoporosis severity (Spearman's r=0.202; p<0.01), suggesting a trend toward milder cases over time (Figure 2). Conversely, a weak but statistically significant negative correlation was found between the year of screening and the treatment status (rs=-0.025; p=0.046) (Figure 3).

**Figure 4:**
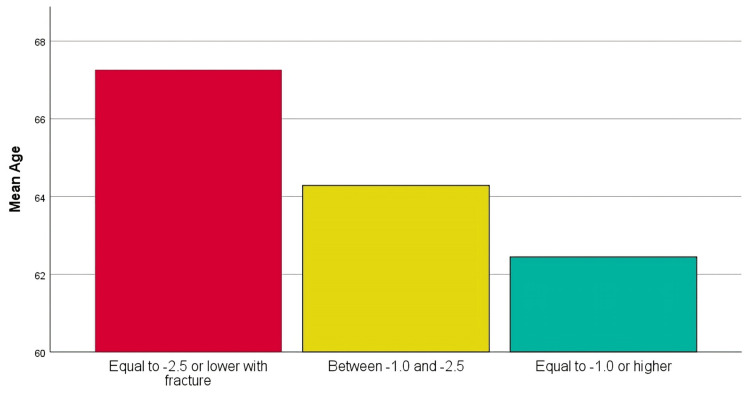
Age and osteoporosis severity An analysis of variance (ANOVA) test was conducted to determine whether there were statistically significant differences in mean age among the groups classified by T-scores. The results revealed a significant difference, with an F-value of 176.225 and a p-value of 0.001, indicating significant differences between the groups

Analysis of variance (ANOVA) showed a significant difference in the mean age across the T-score groups (F=176.225; p<0.001). Patients with osteoporosis and fracture were older (67.3±8.4 years) than those with osteopenia (64.3±7.8 years) or normal BMD (62.5±7.8 years). The effect size was η²=0.089, indicating that approximately 8.9% of the variance in age is explained by differences in T-score categories. This reflects a moderate-to-large effect, suggesting a meaningful relationship between age and osteoporosis severity. Duncan's post hoc analysis confirmed this age-based stratification across severity levels. Similar trends were observed for both men and women (Table 2 and Figure 4).

**Table 2:** Duncan's test using the mean age Duncan's test groups the T-score categories into subsets based on age similarities. Subset 1 includes equal to -1.0 or higher, with the lowest mean age (62.45). Subset 2 includes between -1.0 and -2.5, with a higher mean age (64.28) than Subset 1. Subset 3 includes equal to -2.5 or lower with fracture, with the highest mean age (67.26). This suggests that as T-scores improve, the average age of patients decreases, highlighting the potential relationship between age and osteoporosis severity

T-score	Subset 1	Subset 2	Subset 3
Equal to -1.0 or higher	62.45	-	-
Between -1.0 and -2.5	-	64.28	-
Equal to -2.5 or lower with fracture	-	-	67.26

Patients undergoing treatment were notably older than those not receiving treatment, with ages averaging 69.3±7.2 years compared to 66.0±7.4 years (p<0.001). This trend was observed in both male and female patients, underscoring age as a key factor in determining treatment eligibility (Table 3).

**Table 3:** Age and treatment analysis The table presents the mean±standard deviation of the age distribution for treatment status

Category	No	Yes	t	P-value
Age	66.01±7.420	69.25±7.248	-6.512	0.001

BMI and osteoporosis severity

**Figure 5:**
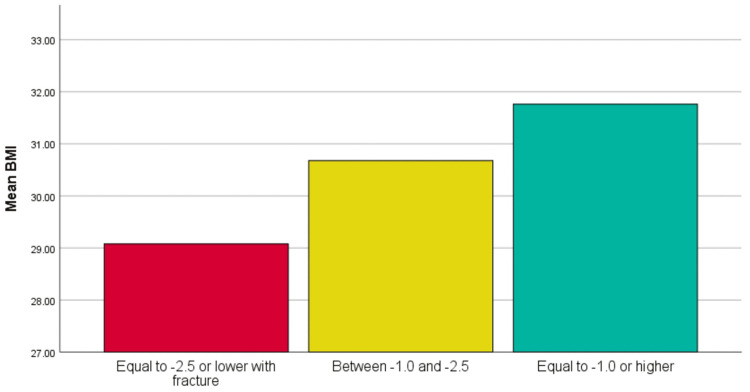
BMI and osteoporosis severity ANOVA revealed significant differences in BMI across T-score categories (F=99.115; p<0.001). Patients with osteoporosis had the lowest BMI (29.1±5.9), while those with normal BMD had the highest (31.8±5.9) BMI, body mass index; ANOVA, analysis of variance; BMD, bone mineral density

ANOVA revealed significant differences in BMI across T-score categories (F=99.115; p<0.001). Patients with osteoporosis had the lowest BMI (29.1±5.9), while those with normal BMD had the highest (31.8±5.9). This trend was true for both sexes and was supported by Duncan's test (Table 4 and Figure 5).

**Table 4:** Duncan's test using mean BMI BMI: body mass index

T-score	Subset 1	Subset 2	Subset 3
Equal to -2.5 or lower with fracture	29.08	-	-
Between -1.0 and -2.5	-	30.68	-
Equal to -1.0 or higher	-	-	31.76

**Figure 6:**
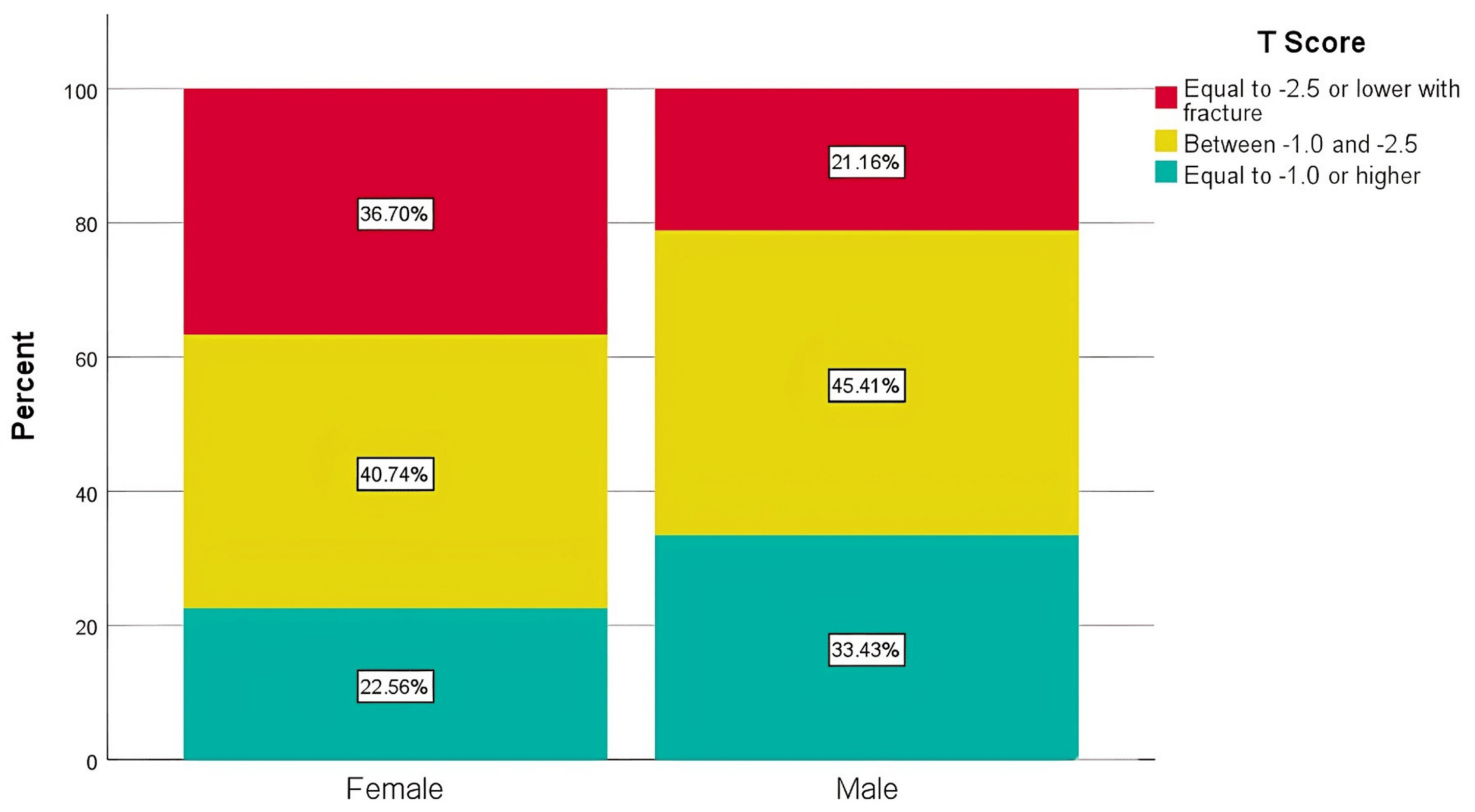
Sex differences in the T-score distribution and osteoporosis severity The chi-square test results indicated a statistically significant variation in T-score distribution across sexes, with a p-value of 0.001, demonstrating meaningful differences between male and female patients

Patients undergoing treatment had a significantly lower BMI compared to those not receiving treatment (28.2±5.5 versus 30.7±6.0; p<0.001). This trend was consistent across both men and women, suggesting a possible association between lower BMI and osteoporosis treatment. In terms of gender-based analysis, there was a notable difference in the distribution of osteoporosis severity between the sexes (p=0.001). Women were more frequently classified in the severe category (36.7% versus 21.2%). However, the treatment rates did not show a significant difference between the sexes (p=0.952) (Figure 6).

The sector-based analysis revealed that the osteoporosis severity distribution varied significantly across geographic sectors (p=0.046), with the northern sector having the most severe cases (Table 5). The observed variation in osteoporosis severity across geographic sectors, particularly the higher severity in the northern sector (p=0.046), may be influenced by several factors. These could include differences in environmental conditions, such as exposure to sunlight and vitamin D synthesis, which are crucial for bone health. Lifestyle factors, such as diet and levels of physical activity, might also contribute, as varying access to nutritious foods and healthcare resources can impact overall bone density. Additionally, genetic predispositions and socioeconomic conditions within the sectors may further explain the disparities in osteoporosis severity. Treatment rates also differed significantly by sector (p=0.003), being the highest in the north (13.6%) and lowest in the south (9.6%) (Table 6).

**Table 5:** Sector differences in T-score distribution and osteoporosis severity The table presents the distribution of T-score classifications by sector. The chi-square test results indicated a statistically significant variation in the T-score distribution across sectors, with a p-value of 0.046, demonstrating meaningful differences between sectors

Sectors	Equal to -2.5 or lower with fracture	Between -1.0 and -2.5	Equal to -1.0 or higher	P-value
East	1,044 (32.88%)	1,342 (42.27%)	789 (24.85%)	0.046
Middle	504 (30.49%)	688 (41.62%)	461 (27.89%)	
North	201 (32.21%)	272 (43.59%)	151 (24.2%)	
South	309 (28.77%)	458 (42.64%)	307 (28.58%)	

**Table 6:** Sector differences in treatment status distribution The table presents the percentage (percent of sector) distribution of the treatment status for sectors. The chi-square test results revealed a statistically significant difference in the treatment status distribution among sectors (p=0.003), indicating significant variation between regions

Category	No	Yes	P-value
East	2,777 (87.46%)	398 (12.54%)	0.003
Middle	1,489 (90.08%)	164 (9.92%)	
North	539 (86.38%)	85 (13.62%)	
South	971 (90.41%)	103 (9.59%)	

## Discussion

This study aimed to investigate the prevalence and management of osteoporosis in Saudi Arabia. Our study in Al-Ahsa, Saudi Arabia, revealed an osteoporosis prevalence rate of 31.54%, which surpasses the global average. This regional rate contrasts with higher prevalence rates in Africa (38.5%) and lower rates in Europe (18.6%), Asia (16.7%), and Australia (13.5%) [5]. Meanwhile, the Americas show a prevalence of 12.4%, and India's rate is 24.7% [5,15]. Aligning with these findings, several studies in Saudi Arabia have reported similar osteoporosis prevalence rates [11-13]. In Riyadh, the prevalence of osteoporosis in the femoral and lumbar spine is 8.2% and 11.8%, respectively, which are mostly found in older women [14]. In Alkhobar, a higher prevalence was observed in men (63.6%) than in women (52.8%), based on lumbar spine T-scores in a younger demographic [12]. These differences may highlight potential genetic, dietary, or healthcare service disparities and emphasize osteoporosis as a significant public health issue in the Middle East. Our study determined that the average age for osteoporosis diagnosis is 68 years for men and 66 years for women, with a prevalence rate of 21.61% for men and 36.7% for women. This finding aligns with the broader literature, which consistently highlights a higher prevalence of osteoporosis among women (35.3% compared to 12.5% in men) [16]. For instance, studies indicate varied prevalence rates in different regions: 10.08% among Chinese women in 2003, 15% among Vietnamese women in 2005, and a significant 24.7% among a diverse Indian cohort [15,17,18]. Similarly, a comprehensive study in China between 2003 and 2015 reported that 25.41% of women and 15.33% of men had osteoporosis [19]. Additionally, research in the eastern Mediterranean found a 24.4% prevalence rate, with 24.4% for women and 20.5% for men, further supporting the observed sex disparity in our findings [11]. These insights underline the urgent need to increase awareness and develop regional interventions specifically targeting aging and female populations. Given the high cost of osteoporosis-related fractures, which amounted to 2.38 billion Saudi Arabian riyal (SAR) in Saudi Arabia in 2019, tackling this health concern is crucial for improving health outcomes and efficiently managing healthcare expenses [20].

Our study discovered an alarming osteoporosis prevalence rate of 31.54% in Al-Ahsa, signifying an urgent need for increased awareness and implementation of targeted screening programs, especially for high-risk populations. Only 11.49% of diagnosed osteoporosis patients in primary healthcare settings received alendronate, highlighting multiple barriers to effective treatment. These may include limited awareness among both patients and providers regarding osteoporosis management guidelines, socioeconomic challenges such as medication cost and access to follow-up care, and poor treatment adherence due to side effects or misunderstanding the long-term importance of therapy. Additionally, systemic gaps in healthcare prioritization of osteoporosis screening and treatment may contribute to undertreatment. Overcoming these obstacles requires a comprehensive strategy focused on education, improved access, and stronger system-level support. This significant gap between diagnosis and treatment highlights the critical need to enhance treatment accessibility and educate the public about the importance of early intervention to prevent osteoporosis-related complications. Age differences in diagnosis were observed, with an average age of 68 years for men and 66 years for women. Notably, the prevalence was significantly higher among women (36.7%) than among men (21.61%). These sex and age disparities underscore the necessity for strategies specifically tailored to effectively manage and prevent osteoporosis among different demographics. Additionally, our findings revealed variations in average BMI values with respect to osteoporosis severity. Patients with a T-score of -2.5 or lower had an average BMI of 29, while those with T-scores between -1.0 and -2.5 exhibited higher average BMIs of 31 and 33, respectively. These findings suggest that BMI has a complex relationship with osteoporosis risk, emphasizing the need for individualized lifestyle and nutritional guidance as part of a comprehensive patient management plan.

In light of these findings, we suggest the implementation of targeted screening programs for populations at high risk, developing educational initiatives for healthcare providers and the public, addressing the treatment gap through improved access to medications and follow-up care, and conducting further research to understand the factors contributing to geographic variations and BMI-osteoporosis relationships. In addition, establishing a comprehensive osteoporosis management program, collaborating with local healthcare authorities on policy development, and implementing long-term follow-up studies will reduce osteoporosis. The study's limitations include potential sampling bias due to the focus on a specific geographic region and the cross-sectional design, which limits causal inferences. Another limitation of this study is the exclusion of many records, which could introduce further selection bias. Additionally, the cross-sectional design limits causal inferences, making it difficult to establish a clear relationship between variables. The reliance on retrospective medical record data may also lead to the omission of important confounding variables, such as diet, exercise, and smoking history. Furthermore, potential measurement errors related to DEXA scanning and inconsistent reporting in medical records could impact the findings. These combined factors suggest that caution is necessary when applying the results to larger populations or drawing definitive conclusions about long-term effects. Despite these limitations, this study has significant implications. The results emphasize the necessity for focused interventions to address identified health disparities, potentially informing policy decisions and resource allocation. Future longitudinal studies could build on these results to establish causal relationships and explore the effectiveness of proposed interventions. This study also underscores the importance of considering socioeconomic factors in health research and practice, indicating the need for a more comprehensive approach to healthcare delivery and public health initiatives.

## Conclusions

This study provides valuable insight into health disparities and their underlying factors. The findings highlight significant disparities in health outcomes across demographic groups, emphasizing the need for targeted interventions and policy changes. Targeted interventions may include strengthening community-based screening programs, clinician education on guideline-based treatment, and incorporating osteoporosis risk assessment into routine check-ups. Policy changes should prioritize expanding insurance coverage for DEXA scans and ensuring equitable access to pharmacologic therapy for at-risk populations. The strengths of this study lie in its comprehensive approach to analyzing multiple health indicators and social determinants. However, future research must address limitations such as the sample size and geographic scope. These findings emphasize the significance of addressing social determinants of health and applying evidence-based strategies to mitigate health inequities. Further research is needed to explore causal relationships, evaluate interventions, and inform policy decisions to improve health outcomes for all population groups.
